# Identifying Programmatic Gaps: Inequities in Harm Reduction Service Utilization among Male and Female Drug Users in Dar es Salaam, Tanzania

**DOI:** 10.1371/journal.pone.0067062

**Published:** 2013-06-25

**Authors:** Barrot H. Lambdin, R. Douglas Bruce, Olivia Chang, Cassian Nyandindi, Norman Sabuni, Sophia Zamudio-Haas, Sheryl McCurdy, Frank Masao, Yovin Ivo, Amani Msami, Omar Ubuguy, Jessie Mbwambo

**Affiliations:** 1 Pangaea Global AIDS Foundation, Oakland, California, United States of America; 2 Department of Global Health, University of Washington, Seattle, Washington, United States of America; 3 School of Medicine, Yale University, New Haven, Connecticut, United States of America; 4 Department of Psychiatry and Mental Health, Muhimbili University of Health and Allied Sciences, Dar es Salaam, Dar es Salaam, Tanzania; 5 Department of Mental Health and Substance Abuse, Ministry of Health and Social Welfare, Dar es Salaam, Dar es Salaam, Tanzania; 6 School of Public Health, University of California, Berkeley, California, United States of America; 7 School of Public Health, University of Texas, Houston, Texas, United States of America; 8 Office of the Prime Minister, Drug Control Commission, Dar es Salaam, Dar es Salaam, Tanzania; Vanderbilt University, United States of America

## Abstract

**Introduction:**

Current estimates suggest an HIV prevalence of 42% among people who inject drugs (PWIDs) in Dar es Salaam, while HIV prevalence is estimated to be 8.8% among the general population in the city. To address the HIV epidemic in this population, the government of Tanzania began establishing HIV prevention, treatment and care services including outreach and medication assisted treatment (MAT) for PWIDs in 2010. We assessed gender inequities in utilization of outreach and MAT services and evaluated differences in HIV risk behaviors between female and male PWIDs.

**Materials and Methods:**

Routine outreach data between December 2010 to mid-August 2012 and baseline data on clients enrolling in methadone from February 2011 to August 2012 were utilized. Binomial regression was used to estimate adjusted relative risk estimates comparing females to males.

**Results:**

From December 2010 to August 2012, 8,578 contacts were made to drug users; among them 1,898 were injectors. A total of 453 injectors were eligible and referred to MAT, of which, 443 enrolled in treatment. However, regarding total outreach contacts, outreach to PWID, referral to MAT and enrollment in MAT, 8% or less of drug users accessing services were women. In contrast, weighted estimations from surveys suggest that 34% of PWIDs are female, and this approximation is similar to recent population size estimations. Overall, 43% of traditional outreach workers conducting outreach with drug users were female. Though reporting higher levels of condom usage, female PWID were more likely to report multiple sex partners, anal sex, commercial sex work and struggle under a higher burden of addiction, mental disorders and abuse.

**Conclusions:**

Services have not been mobilized adequately to address the clear needs of females who inject drugs. A clear and urgent need exists for women-centered strategies that effectively engage female PWID into HIV prevention services.

## Introduction

In the mid-1980s, East Africa became an important stop along international drug trafficking routes thereby introducing heroin in the region [1]. Of the 40–45 tons of opiates that were trafficked into Africa in 2009, most of which entered through East African countries, an estimated 34 tons were consumed in the region, highlighting it not only as a transshipment route but also as a destination for opiate consumption [2]. The United Nations Office of Drugs and Crime estimates there are 1,736,000 heroin users in Africa, 533,000 of which are in Eastern Africa [2]. Heroin use in Dar es Salaam, Tanzania has been extensively documented [1], [3]–[8]. Although mainly consumed by smoking, heroin is increasingly consumed by injection in Tanzania with resultant negative health consequences.

HIV/AIDS has long been linked to injection drug use, a major driver of the HIV epidemic in many countries in Asia, the Middle East and Eastern Europe [9]. Current estimates suggest an HIV prevalence of 8.8% among the general population in Dar es Salaam [10] while HIV prevalence among people who inject drugs (PWIDs) in Dar es Salaam is estimated to be 42% [3]. To address the HIV epidemic in this population, the government of Tanzania began establishing HIV prevention, treatment and care services including outreach and medication assisted treatment (MAT) for PWIDs in 2010.

Concurrently in sub-Saharan Africa, gender inequities lead to a higher burden of HIV among women as 58% of people living with HIV in sub-Saharan Africa are females, and girls and young women aged 15–24 are 2.4 times more likely than their male counterparts to be HIV-positive [11]. In Tanzania, overarching gender inequities lead to higher levels of intimate partner violence [12], HIV risk [13] and HIV burden [10]. Similarly, female PWIDs face higher HIV risk and burden across several fronts [14]–[17]. In Dar es Salaam, approximately 85% of women who inject drugs also engage in sex work, and the mean number of sex partners among sexually active females who inject drugs is 25 in the last 30 days compared to 2 for men [6]. The resources acquired via sex work often allow women to afford larger volumes of better quality heroin resulting in female PWIDs being targets of theft as well as physical and sexual assault by men [1], [6]. This violence drives women out of shared venues, creating insular, hidden communities away from men which further complicates the delivery of risk reduction services and the willingness of women to access services [18]. As a result of these risks, female PWIDs face a higher HIV prevalence than male PWIDs (62% vs. to 28%) [3], which is much higher than the general population (women 10.2% vs. men 7.2% ) [10]. Though methodological concerns exist for gender disaggregation, local agencies report that upwards of 33% of PWIDs in Tanzania are women [19].

In addition to increased risk for HIV, female PWIDs face higher levels of stigma, stronger barriers to accessing services and fewer services that are tailored to their needs [14]–[16], [20]. To assess for programmatic gaps and identify where needs still exist, we assessed inequities in the utilization of MAT and outreach services among male and female drug users and evaluated the differences in HIV risk behaviors of male and female PWIDs utilizing harm reduction services in Dar es Salaam, Tanzania.

## Materials and Methods

### Study Setting

In response to the HIV epidemic among people who inject drugs in Dar es Salaam, the government of Tanzania, namely the Ministry of Health and Social Welfare (MoHSW), Muhimbili University and Hospital of Allied Sciences (MUHAS) and the Drug Control Commission (DCC), in partnership with Pangaea Global AIDS Foundation (Pangaea) and with funding from the US Centers for Disease Control (PEPFAR) launched the first publically funded medication assisted treatment (MAT) program in mainland sub-Saharan Africa at Muhimbili National Hospital. In addition, community-based outreach services for drug users were implemented by this consortium with support from the University of Texas-Houston.

Community-based outreach to drug users was initiated in 2010 through four community-based organizations (CBOs) located in Kinondoni district utilizing three basic approaches: 1) traditional outreach workers who were non-drug using social workers or health professionals; 2) storefront drop-in centers providing information, education and communication (IEC) for HIV risk reduction; syringe cleaning kits; psychosocial services for individuals, families and groups, livelihood and skills training, nutritional support and a venue for 12-step programs; and 3) two mobile units offering HIV testing and counseling (HTC) in drug using communities in Dar es Salaam, Tanzania. Initial outreach was conducted by outreach workers, where clients could be referred to neighborhood storefront drop-in centers for additional care. On a weekly basis, the mobile HTC units coordinated with outreach workers from each of the CBOs to identify locations and times for HTC where the CBOs operate, and outreach workers canvas in the area surrounding the mobile unit. Individuals who tested positive for HIV through mobile HTC units were referred to receive HIV care and treatment at Muhimbili National Hospital or Mwananyamala Regional Hospital.

In February 2011, the first public medication assisted treatment (MAT) clinic on the mainland of sub-Saharan Africa, offering methadone, was launched at Muhimbili National Hospital in Dar es Salaam. Enrollment into the MAT program required referral from a community-based organization. In order to be eligible for MAT, individuals had to exhibit opioid dependence, have evidence of recent drug injection and a positive urine screening for opiates. Before referral to MAT, individuals were also required to attend a series of educational sessions on HIV, STIs, medication adherence, and supportive services provided by CBOs. Once enrolled in MAT, methadone was provided to clients seven days a week at the clinic as a directly observed therapy.

### Study Population

Study subjects were patients who received community-based outreach services from the four CBOs operated by the Tanzanian AIDS Prevention Program in Kinondoni district from December 2010 to mid-August 2012. In addition, study subjects included those who enrolled into the methadone program from February 2011 to August 2012 at Muhimbili National Hospital. In our analysis comparing risk behaviors between male and female PWIDs initiating methadone (see below), we included the first 400 clients with completed baseline assessments.

### Data Sources

The study utilized electronic databases from the four CBOs and the MAT clinic at Muhimbili National Hospital. CBO and clinic management personnel primarily used these databases as tools for clinical management and routine monitoring and evaluation. For community-based outreach services, demographics, drug using behaviors and injection- and sexual-related HIV risk behaviors were collected on all individuals during their first outreach encounter via a brief questionnaire. Clients who enrolled into MAT were asked to complete a comprehensive baseline survey - including modified components of the Addiction Severity Index and Johns Hopkins Symptoms checklist for anxiety and depression - to collect demographic, drug history, legal history, mental health, and HIV risk behavior data. In September of 2012, the databases were collected for the purposes of this evaluation.

### Measures

The patient-level characteristics utilized in our study from the CBO database included demographics: age (in years), sex (male/female) and education level; sex related risk factors: multiple sex partners in the last month (defined as more than 1 casual or regular sex partner), anal sex in the last year, commercial sex work and condom use at last sexual encounter; and injection-related risk factors: shared needles at last injection, shared other equipment at last injection and polysubstance use (alcohol, cocaine, or benzodiazepine). Additionally, the number of outreach contacts with drug users, number of contacts with PWIDs and number of referrals for MAT were also extracted from the CBO database.

The patient characteristics used from the MAT electronic data system included demographics: age (in years), sex (male/female) and education level; sexual risk factors: multiple sex partners in the last 6 months (defined as more than 1 casual or regular sex partner) and regular condom use in the last 6 months (defined as always using a condom during sex); injection risk factors: flashblood - a syringe full of blood drawn back immediately after injection and shared with a companion to inject [8], shared needles at last injection, shared other equipment at last injection, cleaned needles with bleach if sharing at the last injection, polysubstance use (alcohol, cocaine, or benzodiazepine); mental health: high substance dependence (defined as the max score of a 7-point scale); self-reported depression in the last 30 days and self-reported anxiety in the last 30 days; and abuse history: any history of physical abuse and any history of sexual abuse. The number of clients initiating and retained in methadone were also pulled from the MAT electronic data system.

### Statistical Methods

Our primary exposure of interest was the sex (i.e., male vs. female) of outreach and MAT clients. Our outcomes for the study included sexual and injection risk factors from clients accessing community-based outreach and sexual and injection risk factors, mental health and abuse history from medication assisted treatment clients. Binomial regression was used to estimate adjusted relative risk estimates comparing females to males with regards to study outcomes. Potential covariates considered as confounders included education and age. Covariates that were significant at p<0.25 in bivariate analyses were included in the multivariable models to control for confounding.

For estimation of the expected proportion of women PWIDs in outreach and MAT services, we utilized the breakdown from population size estimations and calculated a weighted average of the proportion of female PWID participating in three community-based surveys. The weights were proportional to the total size of the survey. All statistical analyses were conducted using Stata v11.2 (College Station, TX).

Given the use of programmatic data delinked from personal identifiers, this study was approved as nonhuman subjects research by the US Centers for Disease Control and Prevention and Institutional Review Board of Ethical and Independent Review Services. Data will be made freely available upon request.

## Results

### Outreach Clients

A total of 1,898 outreach contacts were made with people who inject drugs between December 2010 and August 2012, 47 of which had missing information regarding the client’s sex (Table 1). Among those with complete information (n = 1,851), 92% of PWIDs receiving outreach were male, and 57% of traditional outreach workers conducting outreach with drug users were male. The average age of PWIDs was 31 years, while males tended to be older on average compared to females (mean age: 31 yrs (males) vs. 29 yrs (females); p<0.001). In addition, females tended to be less educated than their male counterparts (% with more than primary school education: 28% (males) vs. 20% (females); p = 0.017).

**Table 1:** Demographics and Risk Factors of People who Inject Drugs Contacted by Outreach Services.

	PWIDS
	n = 1,898
**Demographics**	
Age (years), n(%)	
≤25	321 (17)
26–35	1134 (62)
36–45	367 (20)
>45	25 (1)
Male, n(%)	1698 (92)
Primary Schooling or Less, n(%)	1365 (72)
**Sexual Risk Factors**	
Multiple Sex Partners in Last Month (regular and/or casual), n(%)	759 (41)
Anal Sex in Last Year, n(%)	139 (8)
Men who Have Sex with Men, n(%)	32 (2)
Performed Commercial Sex Work, n(%)	54 (3)
**Injection Risk Factors**	
Shared Needles at Last Injection, n(%)	378 (26)
Shared other Equipment at Last Injection, n(%)	467 (28)
Polysubstance Use in Last Month, n(%)	555 (29)

Differences between female and male PWIDs with regards to reported sex-related and injection-related HIV risk behaviors are presented in Table 2. Overall, females had a significantly higher likelihood of reporting multiple sex partners (aRR = 1.76; 95% CI: 1.54–2.00); anal sex (aRR = 3.48; 95% CI: 2.45–4.95); and commercial sex work (aRR = 84.00; 95% CI: 36.28–194.25) compared to male PWIDs. However, females were significantly more likely to report having used condoms at last sexual encounter (aRR = 1.22; 95% CI: 1.07–1.39). Males and females reported similar levels of sharing needles (26% (males) vs. 23% (females); p = 0.28) and other equipment (27% (males) vs. 31% (females); p = 0.73), but females were more likely to report polysubstance abuse than males (aRR = 1.28; 95% CI: 1.02–1.61).

**Table 2:** Demographics and Risk Factors of Males and Females who Inject Drugs Contacted by Outreach Services.*

	Male	Female	aRR† (95% CI)	p-value
	n = 1698	n = 153		
**Sexual Risk Factors**				
Multiple Sex Partners in Last Month (regular and/or casual), n(%)	639 (38)	101 (68)	1.76 (1.54, 2.00)	<0.001
Anal Sex in Last Year, n(%)	104 (7)	35 (24)	3.48 (2.45, 4.95)	<0.001
Performed Commercial Sex Work, n(%)	6 (<1)	48 (31)	84.00 (36.28, 194.52)	<0.001
Condom use at last sexual encounter, n(%)	509 (55)	85 (69)	1.22 (1.07, 1.39)	0.004
**Injection Risk Factors**				
Share Needles at Last Injection, n(%)	344 (26)	25 (23)	0.82 (0.57, 1.18)	0.283
Share other Equipment at Last Injection, n(%)	413 (27)	40 (31)	1.05 (0.80, 1.38)	0.733
Polysubstance Use (alcohol, cocaine, or benzodiazepine), n(%)	490 (29)	54 (36)	1.28 (1.02, 1.61)	0.032

* - Missing sex status for 47 PWIDS.

† -Adjusted for age and education; Data Source: Outreach Client Assessment Form.

### Flow of Patients from Outreach to MAT

**Figure 1:**
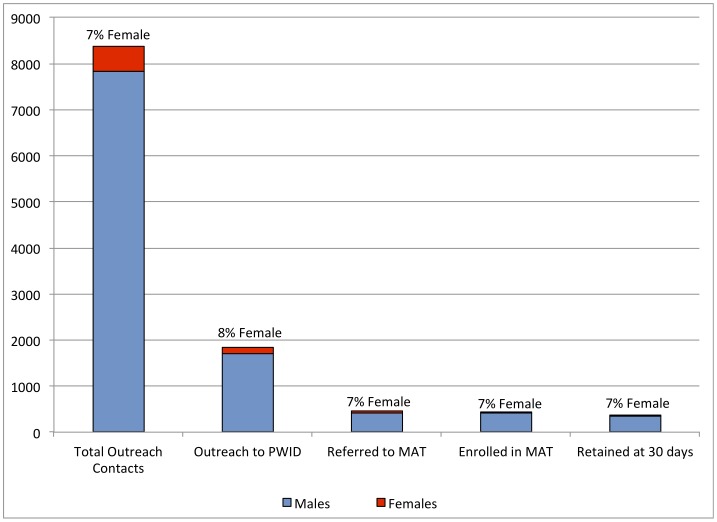
Client Flow from Outreach to Enrollment into Medication Assisted Treatment (MAT).

**Figure 2:**
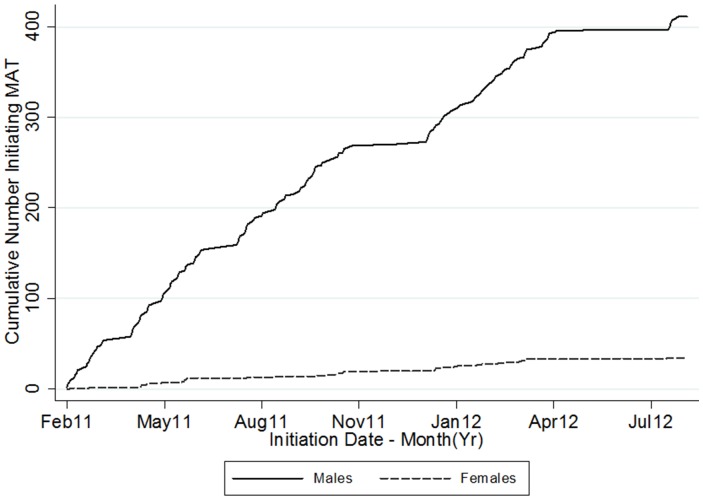
Cumulative Number of Male and Female PWIDs Initiating Medication Assisted Treatment.

Between December 2010 and August 2012, 8,578 outreach contacts were made with drug users, of which 1,898 (22%) were with PWID. Overall, 453 were referred to the MAT clinic at Muhimbili National Hospital. A total of 443 (98%) initiated treatment and 376 were retained in the program at 30 days after initiation (Figure 1). On average, 29 individuals (30 men and 3 women) initiated MAT each month (Figure 2). However, at each of the stages outlined in Figure 1, 8% or less of those accessing services were women. In contrast, from our weighted estimations of three recent surveys [3], [6], [21], we expected 34% of the population of people who inject drugs to be female, and this approximation is similar to population size estimates indicating that 33% of PWIDs are women [19].

### MAT Clients

A total of 400 MAT clients were enrolled between February 2011 and August 2012, with the majority of enrollees (92%) being male. The average age was 34 years at enrollment, while males tended to be older on average compared to females (mean age: 34 yrs (males) vs. 29 yrs (females); p<0.001). Female and male enrollees were not significantly different in education (primary education or lower: 60% (males) vs. 59% (females); p = 0.97), marital status (married: 14% (males) vs. 9% (females); p<0.50), or criminal history (ever arrested: 59% (males) vs. 56% (females); p<0.75).

Differences in HIV risk, mental health and history of abuse between male and female MAT clients are outlined in Table 3. Women had significantly higher likelihood of having multiple sex partners (aRR = 2.42; 95% CI: 1.46–3.98); however, no differences between males and females with regards to injection risk factors were present. With regards to mental health and abuse history, women had a significantly higher likelihood of high substance dependence (aRR = 1.18; 95% CI: 1.05–1.34); depression in the last 30 days (aRR = 1.85; 95% CI: 1.09–3.14); anxiety in the last 30 days (aRR = 1.87; 95% CI: 1.10–3.20); and history of sexual abuse (aRR = 20.21; 95% CI: 3.40–120.13).

**Table 3:** HIV Risk, Mental Health and History of Abuse between Male and Female MAT clients.

	All	Male	Female	aRR† (95% CI)	p-value
	(n = 400)	(n = 368)	(n = 32)		
**Sexual Risk Factors**					
Multiple Sex Partners in last 6 months, n(%)	73 (18)	59 (16)	14 (44)	2.42 (1.46, 3.98)	<0.001
Regular Condom Use during Vaginal Sex in last 6 months, n(%)	89 (37)	77 (36)	12 (41)	1.05 (0.65, 1.70)	0.847
Regular Condom Use during Anal Sex in last 6 months, n(%)	8 (36)	6 (33)	2 (50)	1.09 (0.18, 6.53)	0.922
**Injection Risk Factors**					
Flash-blood, n(%)	38 (10)	35 (10)	3 (10)	0.87 (0.27, 2.77)	0.809
Share Needles at Last Injection, n(%)	56 (14)	48 (13)	8 (25)	1.91 (0.97, 3.75)	0.060
Share other Equipment at Last Injection, n(%)	44 (11)	39 (11)	5 (16)	1.65 (0.71, 3.86)	0.244
Cleaned Needles with Bleach, n(%)	112 (28)	103 (28)	9 (28)	0.95 (0.52, 1.73)	0.869
Polysubstance Use (alcohol, cocaine, or benzodiazepine), n(%)	135 (34)	125 (34)	10 (31)	1.00 (0.59, 1.71)	0.991
**Mental Health**					
High Substance Dependence, n(%)	258 (81)	231 (79)	27 (93)	1.18 (1.05, 1.34)	0.007
Depression in Last 30 Days, n(%)	87 (22)	76 (21)	11 (34)	1.85 (1.09, 3.14)	0.024
Anxiety in Last 30 Days, n(%)	88 (22)	77 (21)	11 (34)	1.87 (1.10, 3.20)	0.021
**History of Abuse**					
Any History of Physical Abuse, n(%)	47 (12)	44 (12)	3 (10)	0.63 (0.20, 2.00)	0.429
Any History of Sexual Abuse, n(%)	5 (1)	2 (<1)	3 (10)	20.21 (3.40, 120.13)	0.001

† -Adjusted for age; Data Source: MAT Program Client Assessment Form.

## Discussion

With data from 1,851 PWIDs receiving community-based outreach services and 400 clients accessing medication assisted treatment at Muhimbili National Hospital, we evaluated for inequities in service utilization between males and females who inject drugs and differences in HIV risk behaviors between male and female PWIDs utilizing services in Dar es Salaam, Tanzania. Compared to population estimates, our assessment identified substantial disparities in women accessing both community-based outreach and MAT services in this study population. Concurrently, female PWIDs face higher levels of sex-related HIV risk behaviors, anxiety, depression and history of sexual abuse. Further implementation science initiatives are critically needed to strengthen harm reduction services to better address the needs of female PWIDs.

The lack of access by women is particularly concerning given the high levels of HIV risk in this population. Female PWIDs face higher levels of sex-related HIV risk behaviors such as anal intercourse placing their sexual risks higher than men who have sex with men since they are engaging in the highest risk sexual behaviors as well as drug injection. Ongoing sexual risks are likely the result of utilizing sex as a means to obtain either drugs or money to support their heroin use or to provide for basic food and shelter. These women are clearly at risk of violence and report higher levels of sexual abuse, with anecdotal reports of physical and sexual violence in this population [7]. Not surprisingly, in this context, the female PWIDs report higher levels of anxiety and depression than male PWIDs. Women were more likely to engage in polysubstance use, and this may be the result of ‘self-medication’ – that is, the use of illicit drugs and alcohol to self-treat the high prevalence of mental disorders as has been suggested in other contexts [22]. Coupled with the risk described above, ongoing substance use entails its own HIV-related risks that continue to place female PWIDs at high risk for HIV as reflected in the latest report estimating HIV prevalence among female PWIDs to be over twice that of male PWIDs [3], [21].

The lack of female engagement in HIV prevention services remains a serious concern for overall HIV prevention in Dar es Salaam because female PWIDs engage in sexual intercourse with non-drug users in an effort to obtain resources. With HIV prevalence over 60% among female PWIDs [21], there remains a real possibility of the HIV epidemic moving from female PWID outside their drug-using social networks and destabilizing the larger HIV epidemic in Dar es Salaam among heterosexual non-drug users. HIV prevention interventions must impact HIV risk behaviors among female PWIDs if HIV incidence is going to decline among PWID in Dar es Salaam.

Programs that have directly addressed the needs specific to women have shown to be effective in other contexts [23]. Currently, community-based outreach and treatment programs in Dar es Salaam do not adequately address the needs of female drug-users including services that address physical and sexual trauma, sexual and reproductive health, economic difficulties and law enforcement discrimination. The low engagement of females also stems from the gendered division of labor that privilege men, where women often have restricted mobility due to domestic responsibilities, particularly with childcare. Females also have fewer opportunities to earn money in the formal sector, leading many to resort to sex work to support their heroin dependency and possibly that of their partners.

Historically, community-based outreach services targeting PWID in Dar es Salaam have utilized traditional outreach workers who were non-drug using social workers or health professionals to outreach to drug using communities. Most of the outreach has focused on reaching drug users in regular hangouts, often referred to as a ‘maskani’, during the day. ‘Maskani’ are open air spaces where groups meet, oftentimes hidden from the general public and police [7]. This approach has resulted in nearly 8,500 outreach contacts with drug users and substantial enrollment of PWIDs into the methadone program during the study period. However, women drug users who also tend to be engaged in sex work are not as embedded in these social networks as men are. Because sex work typically occurs at night, women who engage in sex work do not access those HIV prevention services provided during daytime hours because they typically are sleeping or are involved in domestic responsibilities. Because sex work is illegal in Tanzania, women who engage in sex work are wary of disclosing too much information and engaging with outreach workers or other service providers in order to protect themselves from being arrested. Direct outreach at night, by peer outreach workers and into the more hidden networks of female PWIDs could be strategies that address these obstacles and increase engagement of women within this community.

In addition to impacting outreach contacts, the daily schedule of women who are engaged in sex work or have domestic responsibilities is also an important factor in considering the timing of HIV prevention services at fixed locations. The standard daytime operating hours of the MAT clinic and community-based drop-in centers and their locations present obstacles to many women who do not have sufficient time to travel, especially when relying on public transport, given their other responsibilities. Extending the operating hours of drop-in centers and the MAT clinic to accommodate these schedules is a feasible intervention that could increase women’s engagement. In addition, all or part of the extended hours could specifically cater and have restricted access for female PWIDs, creating a safe space away from men. This strategy could be particularly important to consider as female PWIDs are at increased risk of sexual assault, and physical and sexual violence by male PWIDs has been reported [7]. With regards to MAT services, employing different delivery models such as mobile methadone units at convenient hours could also ameliorate the current time and travel requirements needed to access methadone services.

The principal limitation of our study was due to the observational nature of the research. Although we adjusted for measured patient characteristics to address concerns of confounding, the potential for unmeasured or mismeasured factors to bias our results existed. Regarding the proportion of females expected among people who inject drugs, we pooled data from recent surveys that relied on convenience sampling to build their study population. People who inject drugs are a marginalized population and gathering population-based estimates, disaggregated by sex, can be a challenging endeavor. It is possible that the percentages of female PWIDs expected is an overestimate possibly due to the sampling approaches not yielding a representative sample of the overall population or to female PWIDs experiencing high mortality rates since the surveys were conducted along with a low incidence of new females initiating drug injection. Conversely, it is also important to remember that female PWIDs often face more stigmatization and marginalization than their male counterparts, furthering the difficulty of accurately estimating the proportion of PWIDs who are female as population size estimations often underestimate parts of most at risk populations that tend to be more hidden [24].

### Conclusion

Despite the reality that female PWID are at higher risk of HIV and struggle under a higher burden of polysubstance use, sexual risk taking, anxiety, depression, sexual violence and discrimination, services have not been mobilized adequately to address the clear needs of these women. With a high prevalence of HIV and ongoing sexual risk taking, the HIV epidemic could easily expand outwards from these female PWIDs to their sexual partners, destabilizing the heterosexual HIV epidemic in Dar es Salaam. It is clear, therefore, that an urgent need exists for women-centered HIV prevention services that effectively target female PWID. Successful interventions for this high risk population will yield benefits to the women who are in desperate need of assistance, and to the larger HIV epidemic in Dar es Salaam.
